# Molecular Signatures of Reduced Nerve Toxicity by CeCl_3_ in Phoxim-exposed Silkworm Brains

**DOI:** 10.1038/srep12761

**Published:** 2015-07-31

**Authors:** Binbin Wang, Fanchi Li, Min Ni, Hua Zhang, Kaizun Xu, Jianghai Tian, Jingsheng Hu, Weide Shen, Bing Li

**Affiliations:** 1 School of Basic Medicine and Biological Sciences, Soochow University, Suzhou, Jiangsu 215123, PR China; 2 National Engineering Laboratory for Modern Silk, Soochow University, Suzhou, Jiangsu 215123, PR China

## Abstract

CeCl_3_ can reduce the damage caused by OP pesticides, in this study we used the brain of silkworms to investigate the mechanism of CeCl_3_ effects on pesticide resistance. The results showed that phoxim treatments led to brain damages, swelling and death of neurons, chromatin condensation, and mitochondrial damage. Normal nerve conduction was severely affected by phoxim treatments, as revealed by: increases in the contents of neurotransmitters Glu, NO, and ACh by 63.65%, 61.14%, and 98.54%, respectively; decreases in the contents of 5-HT and DA by 53.19% and 43.71%, respectively; reductions in the activities of Na^+^/K^+^-ATPase, Ca^2+^/Mg^2+^-ATPase, and AChE by 85.27%, 85.63%, and 85.63%, respectively; and increase in the activity of TNOS by 22.33%. CeCl_3_ pretreatment can significantly reduce such damages. Results of DGE and qRT-PCR indicated that CeCl_3_ treatments significantly upregulated the expression levels of *CYP4G*23, *cyt-b*5, *GSTs-σ*1, *ace*1, *esterase-FE*4, and *β-esterase* 2. Overall, phoxim treatments cause nerve tissue lesions, neuron death, and nerve conduction hindrance, but CeCl_3_ pretreatments can promote the expression of phoxim resistance-related genes in silkworm brains to reduce phoxim-induced damages. Our study provides a potential new method to improve the resistance of silkworms against OP pesticides.

Silkworm *(Bombyx mori, B. mori)* is an important economic insect and a model species for *Lepidoptera*, in China it produces more than 80% of raw silk of the world^1^. It has been domesticated for about 5,700 years in China. However, *B. mori* became very sensitive to pesticides and other chemicals because of long-term indoor breeding and limited exposure to the outside environment. Therefore, it is also used as a model insect to study the toxicology of pesticides and pest control and serves as an environmental indicator. Organophosphorus (OP) based pesticides are one of the most widely used pesticides. The main mechanism of action of OP pesticides is to irreversibly bind acetylcholinesterase (AChE) and inhibit its activity, which leads to the accumulation of neurotransmitter acetyl choline (ACh) in synaptic clefts then insect convulsion and eventual death, because nervous excitement cannot be terminated^2^,^3^. Wide use of pesticides has significantly improved agriculture productions, but the pollution from them to the environment has also become a big problem. In China, pesticide-contaminated mulberry causes up to 30% loss in silk industry every year^4^. Therefore, how to reduce such losses has become a popular topic in recent years. Previous studies have shown that titanium dioxide can reduce the damage caused by OP pesticide phoxim, and the mechanism has been explored^5^.

Rare earth elements (REEs) are widely used in various industries due to their diverse physical and chemical properties^6^, including agriculture and pharmaceutical industry, for example Cerium can relieves the inhibition of chlorophyll biosynthesis of maize caused by magnesium deficiency^7^, Cerium can increase taxol accumulation of Taxus cuspidata cells^8^. Recent study showed that CeCl_3_ pretreatment could increase the growth and survival rate of *B. mori* under phoxim-induced toxicity by increasing antioxidant capacity and improving protein and carbohydrate metabolism^9^. CeCl_3_ pretreatment could decrease oxidative damage of *B. mori* caused by nucleopolyhedrovirus infection via increasing antioxidant capacity^10^. CeCl_3_ pretreatment could alter the gene expression pattern and relieves the damages in the midgut of *B. mori* caused by phoxim^11^. CeCl_3_ can relieves the damages caused by phoxim, protect *B. mori* silk gland and remit the reduction in body weight and cocooning rate through changing gene expression patterns and reducing oxidative stress^12^.

Brain is the main part of central nervous system and the important target of phoxim. Whether CeCl_3_ can relieve the brain damage in *B. mori* caused by phoxim is still unknown. In this study, brain was used as the study object to further explore the mechanism of relief from toxic symptoms caused by phoxim under CeCl_3_ pretreatment.

## Results

### Histopathological evaluation of brain

**Figure 1:**
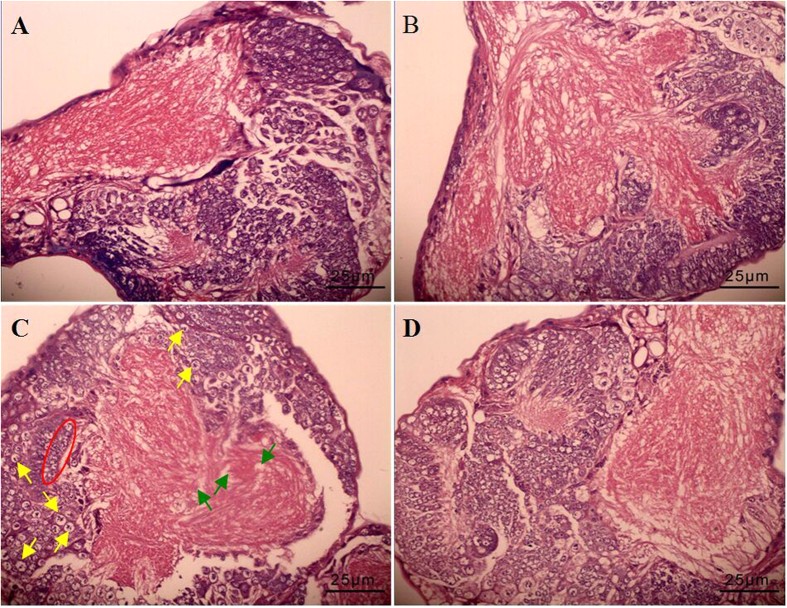
Histopathology of the brain tissue in fifth-instar larvae after phoxim exposure. (**A**) Control; (**B**) CeCl_3_; (**C**) phoxim; (**D**) CeCl_3_ + phoxim. Green arrows indicate breakage of nerve fibers, yellow arrows indicate cell swollen and death, red box indicate cell death.

As shown by the histological photomicrographs of *B. mori* larval brain sections in Fig. 1, both control group (Fig. 1A) and CeCl_3_-treated group (Fig. 1B) had no abnormal pathological changes, with spherical glial cells, fusiform neurons cells and nerve fiber tracts showing clear and complete structures. In the phoxim-exposed group, we observed that glial cells and neurons were swollen, along with loss in cell contents, nucleus fragmentation, cell death, nerve fibers breakage, protein aggregation, and adipose degeneration (Fig. 1C). As a contrast, the CeCl_3_ + phoxim-treated group did not show such pathological changes (Fig. 1D). These results demonstrated that phoxim exposure caused brain damages, while CeCl_3_ treatments were able to reduce them.

### Brain ultrastructure evaluation

**Figure 2:**
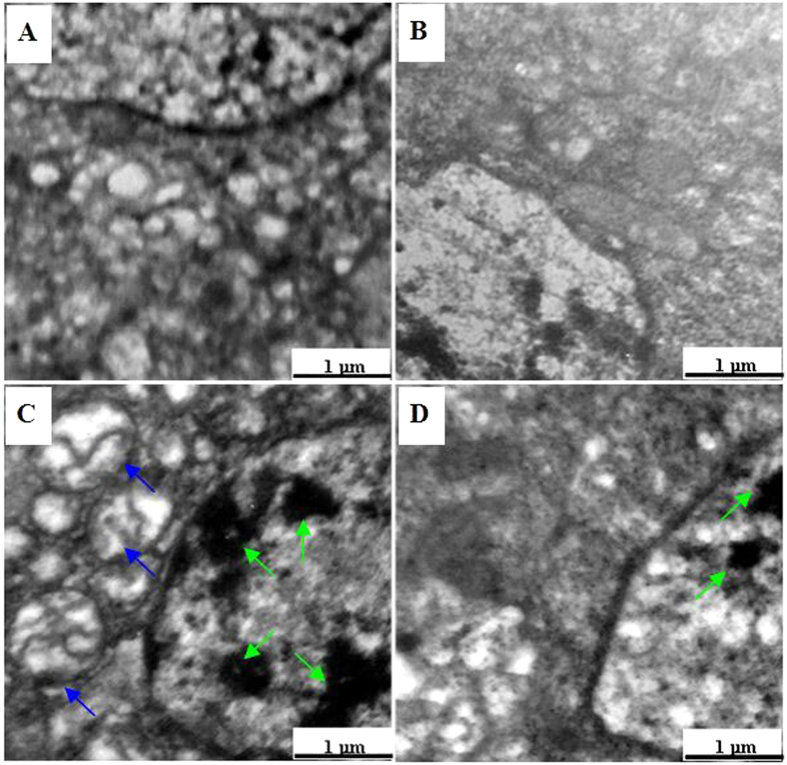
Ultrastructure of the brain tissue in fifth-instar larvae after phoxim exposure. (**A**) Control; (**B**) CeCl_3_; (**C**) phoxim; (**D**) CeCl_3_ + phoxim. Green arrows indicate karyopyknosis and chromatin marginalization, blue arrows show mitochondria swelling and became deformed, crest broken.

Changes in brain ultrastructure in silkworms were presented in Fig. 2. Both the control group (Fig. 2A) and CeCl_3_-treated group (Fig. 2B) had normal structures, along with evenly distributed nuclear chromatin, integral mitochondria structure, clear mitochondria ridges. The phoxim-treated group (Fig. 2C), on the other hand, showed karyopyknosis, chromatin marginalization, swelling mitochondria, vacuolar degeneration, rough surfaced endoplasmic reticulum, and extended golgi’s apparatus, while the CeCl_3_ + phoxim-treated group had reduced phoxim-induced damages (Fig. 2D).

### Neurotransmitter contents and enzyme activities in the brain

Contents of neurotransmitters and activities of related enzymes were analyzed in order to investigate phoxim-induced nerve damages in the brain of fifth instar larvae. As shown in Table 1, in the control group and CeCl_3_-treated group, the contents of neurotransmitters Glutamate (Glu), nitric oxide (NO), ACh, 5-hydroxytryptamine (5-HT), and dopamine (DA) did not change significantly. However, in the phoxim-treated group, contents of Glu, NO and ACh were all significantly increased, by 63.65%, 61.14%, and 98.54%, respectively (*P* < 0.01), while the 5-HT and DA contents were significantly decreased by 53.19% and 43.71%, respectively (*P* < 0.01) (Table 1). These results indicated that CeCl_3_ treatments significantly alleviated phoxim-induced damages. In addition, the CeCl_3_-treated group did not show significant differences in the activities of Na^+^/K^+^-ATPase, Ca^2+^/Mg^2+^-ATPase, AChE, and total nitric oxide synthase (TNOS) from those of the control group; as a contrast, phoxim treatments significantly reduced the activities of Na^+^/K^+^-ATPase, Ca^2+^/Mg^2+^-ATPase, and AChE by 85.27%, 85.63%, and 85.63%, respectively (all *P* < 0.01), while significantly increasing the activity of TNOS by 22.33% (*P* < 0.01) (Table 2); these results demonstrated that phoxim can significantly interfere with the nerve conduction in the brain of silkworm fifth instar larvae, and that CeCl_3_ can relieve such interference.

### High throughput sequencing

**Figure 3:**
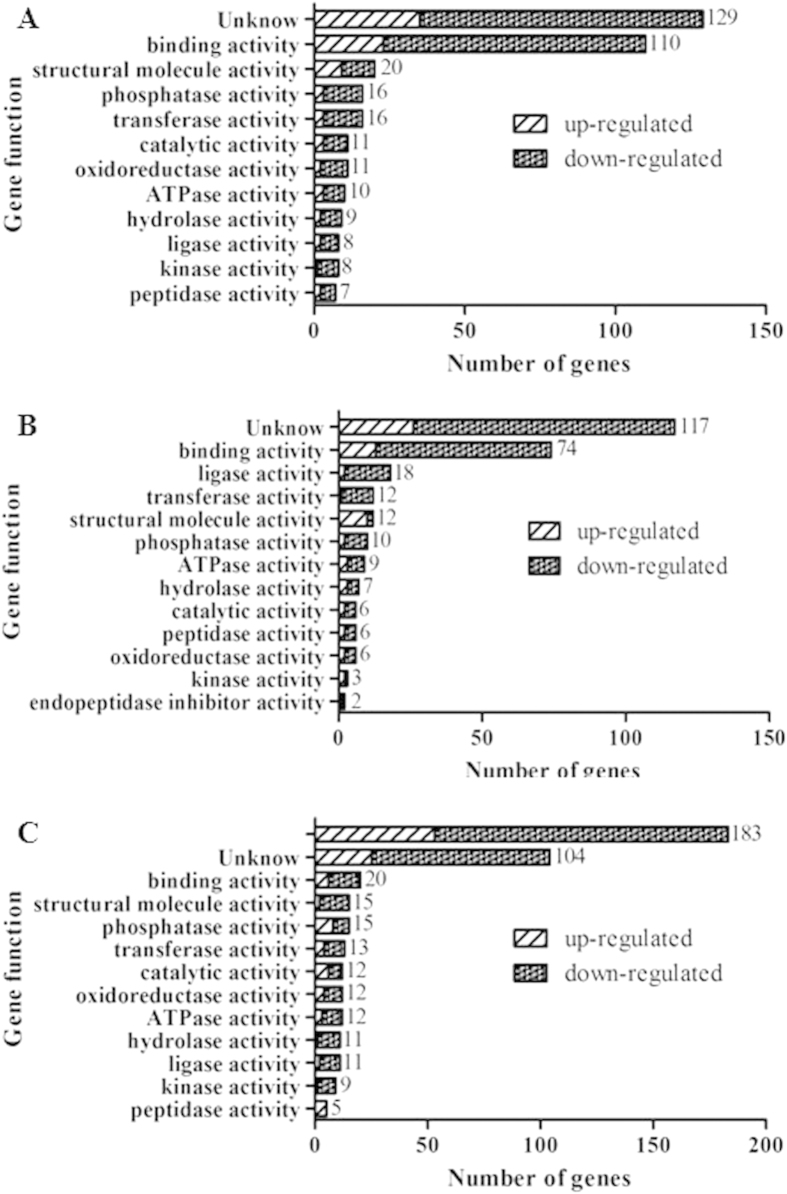
Significantly changed genes classify. (**A**) Functional categorization of 355 genes which significantly altered by CeCl_3_ pretreatment; (**B**) Functional categorization of 282 genes which significantly altered by phoxim exposure; (**C**) Functional categorization of 422 genes which significantly altered by CeCl_3_ + phoxim treatment; Genes were classified based on the GO function.

In order to investigate the changes of gene expression pattern in brain caused by CeCl_3_’s and phoxim treatment, we used the digital gene expression (DGE) method to detect the differences in gene expression. The CeCl_3_-treatment group (Fig. 3A), the phoxim-exposure group (Fig. 3B), and the CeCl_3_ + phoxim group (Fig. 3C) had 355, 282, and 422 genes that were significantly changed compared with the control group, among which 88, 69 and 120 genes were upregulated, respectively, and 267, 213 and 302 genes were downregulated, respectively. According to the results from Gene Ontology (GO) function analysis, those genes are divided into the following groups: binding activity, structural molecule activity, ligase activity, transferase activity, phosphatase activity, ATPase activity, hydrolase activity, oxidoreductase activity, peptidase activity, catalytic activity, kinase activity, and endopeptidase inhibitor activity. The significantly changed genes that have been known in each group were listed in Table S1.

### qRT-PCR verification of gene expression changes

A number of genes that showed significantly different expression patterns in DGE assay were selected for qRT-PCR verification due to their functions. These genes are involved in nerve conduction, pesticide metabolism, apoptosis, and oxidative stress. With CeCl_3_ + phoxim treatments, the expression levels of cytochrome P450 family 4G23 (*CYP4G*23), cytochrome b5 (*cyt-b*5), *GSTs-σ*1, acetylcholinesterase type 1 gene (*ace*1), *esterase-FE*4, *β-esterase* 2, and catalase (*CAT*) were increased 12.583, 8.623, 16.462, 5.843, 6.714, 2.583, and 2.793 fold, respectively. With CeCl_3_ treatment, *CYP4G*23 was increased by 3.472 fold, while phoxim treatment increased *CYP4G*23’s level by 6.363 fold, ace1’s level by 19.453 fold, and *esterase-FE*4’s level by 3.671 fold (Table 3). The results indicated that CeCl_3_ changes the gene expression response of silkworm larva’s brains to phoxim, consistent with the DGE results.

## Discussion

OP pesticides are widely used in China. Application of large quantity of pesticides can certainly control varied types of pests, but it is also polluting the environment. Silkworms are very sensitive to pesticides, thus pesticide pollution causes serious losses to China’s sericulture^4^. How to increase silkworm’s pesticide resistance to mitigate economic losses has become an urgent problem. In this study, we found that addition of an appropriate amount of CeCl_3_ onto mulberry leaves can reduce phoxim-induced damages. The mechanism of CeCl_3_’s effects on phoxim-induced damages was also investigated in order to seek for methods to better improve silkworm’s resistance to pesticides.

The major damage of phoxim comes from its destroy to nervous^3^. We also performed detailed investigation on silkworm brains after phoxim treatments. Phoxim caused serious brain tissue damages and cell death (Fig. 1). Analysis of ultrastructure revealed severely damaged endoplasmic reticulum, golgi apparatus, and mitochondria (Fig. 2). At the same time, the contents of neurotransmitters Glu, NO, and ACh were increased significantly, along with significantly decreased contents of 5-HT and DA and significantly inhibited activities of Na^+^/K^+^-ATPase, Ca^2+^/Mg^2+^-ATPase, and AChE. Na^+^/K^+^-ATPase, Ca^2+^ and Mg^2+^ ion, AChE play an important role in conduction of action potential, ACh transport, ACh degradation respectively^13^,^14^. When CeCl_3_ was given to the larvae, such changes were significantly relieved.

Cytochrome P450, GSTs, and esterase are essential parts of the detoxification system of insects^15^,^16^. In this study, we observed increased expression of *CYP4G*23, a member of *CYP*4 families which are well consistent with their predicted role in xenobiotic metabolism^17^, indicating that phoxim poisoning induces *CYP4G*23 as a response in larval brain, such response was even stronger in the CeCl_3_ pretreatment group (Table 3). *Cyt-b*5, an essential molecule in P450-mediated detoxification^18^, was significant increase in the group of CeCl_3_ + phoxim (Table 3). GSTs have multiple subunits and play important roles in the biotransformation of exogenous compounds, drug metabolism, and the protection against peroxidation damages. Therefore, increases in the expression of GSTs are one of the major reasons of enhanced pesticide resistance^19^. In this study, we found that the subunit *GSTs-σ*1’s expression was significantly reduced by phoxim but significantly increased by CeCl_3_ pretreatments (Table 3), indicating that CeCl_3_ promotes the response to phoxim through increasing GSTs contents. Esterases exert their detoxification effects by direct binding to or hydrolysis of pesticides, and many studies have reported that overexpression of esterases can increase insects’ resistance to pesticides^20^,^21^. The overexpression of β-esterase increase the resistance against deltamethrin in *Rhipicephalus (Boophilus) microplus*^22^. The expression quantity of esterase-FE4 is positive correlation to acephate in *Myzus persicae* (SULZER)^23^. In the present study, CeCl_3_ treatments increased the expression levels of AChE, β-esterase 2, and esterase-FE4 (Table 3), which may be one of the reasons for increased pesticide resistance of silkworms. We also observed a significant increase in the expression of GPI anchor after CeCl_3_ treatments (Table S1), AChE is anchored to neuron membrane through GPI anchor. Previous study has indicated that increased AChE anchoring leads to improved pesticide resistance^24^.

Mitochondria are not only the energy factories of eukaryotic cells but also the primary targets of toxicity after pesticides enter cells^25^,^26^. Pesticides destroy mitochondrial structure to release free radicals, which damage DNA, interfere signal transduction, and affect the expression of apoptosis-related genes that leads to apoptosis^27^,^28^,^29^,^30^. In the present study, phoxim caused serious damages to the mitochondria of silkworm larval brains, cell abnormalities, and even apoptosis, and all these damages were alleviated by CeCl_3_ pretreatments (Figs 1 and 2). Results from high-throughput sequencing indicated that CeCl_3_ treatment can increase the expression of CAT, xanthine dehydrogenase (XDH), and serine-pyruvate aminotransferase (AGT) (Table 3, Table S1), these enzymes reduce mitochondrial damages through scavenging free radicals.

Cerium ion has some special features, including functioning as an antibiotic^31^ and cleaving the phosphodiester of DNA^32^. Wang *et al.* attempted to hydrolyze the phosphodiester of OP using Cerium complexes with saccharides and revealed that the complexes were able to degrade methamidophos, omethoate and dimethoate^33^. These complexes can also reduce the chlorpyrifos and parathion residue in jujube^34^. We speculate that absorbed Cerium ion may hydrolyze phoxim to some extent and protect the silkworm, which needs further studies to confirm.

In this study, we found that CeCl_3_ pretreatment could reduce phoxim-induced nerve damages. CeCl_3_ enhanced the expression of detoxification enzymes, esterases, P450s, and GSTs, which participate in phoxim metabolism; CeCl_3_ could also upregualte the genes related to free radical clearance and decrease the expression of apoptosis genes to protect neurons in the brain. As a result, CeCl_3_ may enhance phoxim metabolism in silkworms to protect their nerve systems. This study illustrated the potential mechanisms of CeCl_3_’s effect to enhance phoxim metabolism in silkworm brains and provided a theoretical basis to use CeCl_3_ as an additive to improve silkworms’ pesticide tolerance in sericulture.

## Methods

### Insect and chemicals

The larvae of *B. mori* (strain: Qiufeng × baiyu) maintained in our laboratory were reared at 27 ± 2 °C on mulberry leaves under a 12 h light/12 h dark cycle.

Phoxim was purchased from Sigma-Aldrich (USA). CeCl_3_ (analytical grade, 99.99%) was purchased from Shanghai Chem. Co. (China).

### Treatment and brain tissue collection

Phoxim stock solution was prepared by 10 × dilution using acetone. For the treatment, phoxim stock solution was dissolved in water to obtain a concentration of 4 μg/mL. The lethal concentration (*LC*_50_) of phoxim in *B. mori* was 7.86 μg/mL, and at 4 μg/mL, *B. mori* showed poisoning symptoms without death^1^,^4^. In a pre-experiment, different concentrations (0.1, 0.2, 0.5, 1.0, and 1.5 mg/L) of CeCl_3_ was used, which were administered to fifth-instarlarvae, the optimum concentration of CeCl_3_ was 0.5 mg/L for growth of these larvae^10^. *B. mori* larvae were feed with CeCl_3_-treated leaves (leaves were dipped in 0.5 mg/L CeCl_3_ solution for 1 min and dried in the air) and normal leaves respectively three times a day until the 2^nd^ day of fifth-instar. Then a part of the larvae of these two groups were feed with phoxim-treated leaves (leaves were dipped in 4 μg/mL phoxim for 1 min and dried in the air). Each treatment was performed three times. Forty-eight hours after phoxim treatments, 100 fifth-instar larvae was selected randomly from each group to collect brain tissues that were frozen at −80 °C for further study.

### Histopathological evaluation of brain

All histopathological examinations were performed using the follow laboratory procedures. Five brains from the larvae of each group were embedded in paraffin, sliced (5 μm thickness), placed onto glass slides, and stained with hematoxylin-eosin for 15 min. Stained samples were observed and photographed using an optical microscope (Nikon U-III Multi-point Sensor System, Japan).

### Observation of brain ultrastructure

Five larvae’s brain tissues of each group were fixed in freshly prepared 0.1 M sodium cacodylate buffer with 2.5% glutaraldehyde and 2% formaldehyde, before being treated at 4 °C with 1% osmium tetroxide in 50 mM sodium cacodylate (pH 7.2-7.4) for 2 h. Staining was performed overnight with 0.5% aqueous uranyl acetate. After serial dehydration with ethanol (75, 85, 95, and 100%), the specimens were embedded in Epon 812 and sliced. Ultrathin sections were treated with uranyl acetate and lead citrate, and observed with a HITACHI H600 TEM (HITACHI Co., Japan). The damages of brain was determined by observing the changes in nuclear morphology, (e.g., chromatin condensation and fragmentation).

### Assay of enzymatic activities

To determine enzymatic activities, brain tissues were homogenized in 0.15 M NaCl. The homogenate of brains was centrifuged at 3,000 g for 15 min at 4 °C, A portion of supernatant was used to measure the activities of different enzymes. The activities of AChE, Ca^2+^-ATPase, Ca^2+^/Mg^2+^-ATPase, Na^+^/K^+^-ATPase, and TNOS in the brain were measured spectrophotometrically with commercial kits (Nanjing Jiancheng Bioengineering Institute, China), targeting the oxidation of oxyhaemoglobin to methaemoglobin by nitric oxide.

### Measurements of neurochemicals

The homogenate of brains was centrifuged at 12,000 g for 20 min at 4 °C. The concentrations of DA, 5-HT, and ACh were measured spectrophotometrically with commercially kits (Nanjing Jiancheng Bioengineering Institute, China).

Glu concentrations were measured using commercial kits (Nanjing Jiancheng Bioengineering Institute, China), and standard curves were generated by using standard Glu stock solutions. Sample Glu levels were detected using a spectrophotometer at 340 nm and expressed as μmol/g prot. The concentration of NO in the brain was measured using a commercial kit (Nanjing Jiancheng Bioengineering Institute, China). The OD value was determined by using a spectrophotometer (U-3010, Hitachi, Japan). NO results were read with OD values at 550 nm. The results were calculated using the following formula: NO (μmol/L) = (Asample – Ablank)/(Astandard – Ablank) × 20 (μmol/L).

### Total RNA isolation

Trizol reagent was used to extract the total RNA from brain samples (Takara, Dalian China) and treated with DNase to remove potential genomic DNA contamination. The quality of the RNA was quantitated spectrophotometrically at 260 and 280 nm.

### Digital gene expression library preparation and sequencing

For RNA library construction and deep sequencing, equal quantities of brain RNA samples (n = 3) were pooled for the control group and the treatment group, respectively. Approximately 6 μg of RNA representing each group was submitted to Solexa (now Illumina Inc.) for sequencing. Detailed methodology was according to the method described by Gu *et al.*^35^.

### qRT-PCR analysis

The specific primers for the 7 genes of interest are listed in Table 4. The internal reference gene was *actin*3. qRT-PCR was performed using the 7500 Real-time PCR System (ABI) with SYBR Premix Ex *Taq*TM (Takara, Japan) according to the manufacturer’s instructions. The qRT-PCR analysis was carried out following the method described in the studies of Peng *et al.* and Wang *et al.*^4^,^36^.

## Additional Information

**How to cite this article**: Wang, B. *et al.* Molecular Signatures of Reduced Nerve Toxicity by CeCl_3_ in Phoxim-exposed Silkworm Brains. *Sci. Rep.* **5**, 12761; doi: 10.1038/srep12761 (2015).

## Supplementary Material

Supplementary Information

## Figures and Tables

**Table 1:** Effects of phoxim and CeCl_3_ on neurotransmitter contents in brain of silkworm.

	**GLu (μmol/g protein)**	**NO (μmol/g protein)**	**Ach (μg/g tissue)**	**5-HT (μg/g tissue)**	**DA (μg/g tissue)**
Control	55.35 ± 1.22	72.32 ± 1.49	10.26 ± 1.91	23.35 ± 2.91	20.11 ± 1.83
CeCl_3_	52.49 ± 1.26	67.53 ± 1.17	8.55 ± 1.39	25.82 ± 3.03	22.25 ± 2.15
Phoxim	90.58 ± 1.82**	116.54 ± 2.31**	20.37 ± 2.86**	10.93 ± 0.91**	11.32 ± 1.28**
*Ce* *+* *Phoxim*	67.12 ± 1.45*	79.89 ± 1.85	12.21 ± 1.66	20.71 ± 1.21	18.9 ± 1.69

*P < 0.05, and **P < 0.01. Values represent means ± SD (*N* = 5).

**Table 2:** Effects of phoxim and CeCl_3_ on enzyme activities in 5th-instar larva brain of silkworm.

	**Na**^**+**^**/K**^**+**^**-ATPase (U/mg protein·min)**	**Ca**^**2+**^**/Mg**^**2+**^**-ATPase (U/mg protein·min)**	**AchE (U/mg protein·min)**	**TNOS (U/mg protein·min)**
Control	2.58 ± 0.31	1.74 ± 0.12	1.74 ± 0.19	7.22 ± 0.49
CeCl_3_	3.39 ± 0.39*	1.82 ± 0.17	1.95 ± 0.21	6.73 ± 0.38
Phoxim	0.38 ± 0.08***	0.23 ± 0.07***	0.25 ± 0.06***	11.64 ± 1.15**
*Ce + Phoxim*	1.90 ± 0.13*	1.42 ± 0.15*	1.48 ± 0.15*	7.98 ± 0.61

*P < 0.05, **P < 0.01, and ***P < 0.001. Values represent means ± SD (*N* = 5).

**Table 3:** Comparison between fold-difference with qRT-PCR results and DGE assay in each group.

**Gene**	**Treatment**					
	**CeCl**_**3**_**/Control**		**phoxim/Control**		**CeCl**_**3**_** + phoxim/Control**	
	**qRT-PCR (Fold)**	**DGE (log_2_ value)**	**qRT-PCR (Fold)**	**DGE (log_2_ value)**	**qRT-PCR (Fold)**	**DGE (log_2_ value)**
*CYP4G*23	3.472***	2.537	6.363***	4.228	12.583***	5.755
*Cyt-b*5	1.047	No difference	0.945	No difference	8.623***	4.854
*GSTs-σ*1	0.835	−0.408	0.262**	–2.827	16.462***	4.002
*ace*1	0.908	0.072	19.453***	0.956	5.843***	1.059
*esterase-FE*4	1.142	No difference	2.671**	5.365	6.714***	5.487
*β-esterase* 2	0.722*	No difference	0.869	No difference	2.583**	2.209
*CAT*	1.106	No difference	1.028	No difference	2.793**	2.998

*P < 0.05, **P < 0.01, and ***P < 0.001. Values represent means ± SD (*N* = 5).

**Table 4:** The detail sequences of the primers for the genes selected for qRT-PCR analysis.

**Gene**	**Primer sequences (5’-3’)**	**Product size (bp)**
*CYP4G*23	TATTGACACGCCCATAAAG GTAGGAGATTGGCTGTTGC	119
*Cyt-b*5	CTGAAGCCAAGGACCGCAC TATCGCCACCGCCAGGATG	135
*GSTs-σ*1	TGGAGTTCCTGCATGATAT CTTTGTTCGCTTCAACTATC	150
*ace*1	CTCCAGTTCAGTTGGTCGTG ACAGTGCTGTGCCTGTAAGC	197
*EST-FE*4	GTGGCACTCTTGCGTTGGG CCTTCGTGGTGTCTGATAG	130
*β-esterase* 2	ACCCAATAACATACCGACTG CACAAAGGCAATGAACCC	135
*CAT*	AACTTCTCCCAAGCGACAG GAATAAACGCAGCAGCATC	109
*Action*3	CGGCTACTCGTTCACTACC CCGTCGGGAAGTTCGTAAG	147
