# Supplementary material for: Molecular Signatures of Reduced Nerve Toxicity by CeCl3 in Phoxim-exposed Silkworm Brains
Source: Sci Rep. 2015 Jul 31;5:12761. doi: 10.1038/srep12761 (PMC4521201; doi:10.1038/srep12761)
Supplement: Supplementary Information [file srep12761-s1.doc]

**Molecular Signatures of Reduced Nerve Toxicity by CeCl_3_ in Phoxim-exposed Silkworm Brains**

Binbin Wang ^1,*^, Fanchi Li ^1,*^, Min Ni ^1,*^, Hua Zhang ^1^, Kaizun Xu ^1^, Jianghai Tian ^1^, Jingsheng Hu ^1^, Weide Shen ^1,2^ & Bing Li ^1,2^

^1^ School of Basic Medicine and Biological Sciences, Soochow University, Suzhou, Jiangsu 215123, PR China, ^2^ National Engineering Laboratory for Modern Silk, Soochow University, Suzhou, Jiangsu 215123, PR China

Correspondence and requests for materials should be addressed to Bing. Li ([lib@suda.edu.cn](mailto:lib@suda.edu.cn))

^*^These authors contributed equally to this study.

**Table S1. Genes that significantly changed by DGE assay in each group**

| Name | Sequence ID | CeCl_3_/Control (log2 value) | phoxim/Control (log2 value) | CeCl_3_+phoxim/Control (log2 value) |
| --- | --- | --- | --- | --- |
| sex-specific storage-protein 1 precursor | GI: 169234936 | No difference | 2.942 | 6.406 |
| alanine-glyoxylate aminotransferase | GI: 512928590 | No difference | No difference | 6.098 |
| MFB-03L16 | GI: 346714807 | 1.997 | 1.174 | 5.901 |
| aliphatic nitrilase | GI: 284813565 | No difference | 2.478 | 5.767 |
| CYP4G23 | GI: 433338895 | 2.537 | 4.228 | 5.755 |
| esterase -FE4 | GI: 339283872 | No difference | 5.365 | 5.487 |
| Heat shock protein 25.4 precursor | GI: 169646838 | No difference | No difference | 5.422 |
| heat shock protein 25.4 precursor | GI: 169646838 | No difference | No difference | 5.421 |
| odorant binding protein LOC100301495 precursor | GI: 237648972 | 3.2847 | 1.3785 | 5.237 |
| tryptophan 2,3-dioxygenase-like | GI: 512891302 | No difference | No difference | 5.042 |
| low molecular 30 kDa lipoprotein PBMHP-6 precursor | GI: 112984502 | No difference | 1.6757 | 4.860 |
| Cyt-b5 | GI: 164448654 | No difference | No difference | 4.854 |
| juvenile hormone binding protein brP-2095 precursor | GI: 112983390 | 4.528 | 5.1341 | 4.852 |
| serine protease inhibitor 7 precursor | GI: 226342878 | No difference | No difference | 4.786 |
| GMC beta2 | GI: 395805236 | No difference | -11.355 | 4.774 |
| ommochrome-binding protein-like | GI: 512934697 | No difference | 2.087 | 4.637 |
| fungal protease inhibitor F precursor | GI: 112983466 | 3.281 | 3.2423 | 4.624 |
| chemosensory protein 7 precursor | GI: 112983052 | No difference | 1.379 | 4.284 |
| vitellogenic carboxypeptidase-like | GI: 512917901 | 3.711 | 3.317 | 4.179 |
| promoting protein precursor | GI: 112984526 | No difference | 1.139 | 4.161 |
| 30kDa protein | GI: 312597592 | No difference | 2.049 | 4.075 |
| GSTs-σ1 | GI: 112983028 | -0.408 | -2.827 | 4.002 |
| 30K protein 6 | GI: 379046486 | No difference | No difference | 3.911 |
| 30K protein 7 | GI: 379046488 | No difference | No difference | 3.785 |
| seroin 1 precursor | GI: 112983118 | 2.874 | No difference | 3.805 |
| C-type lectin 11 precursor | GI: 112983022 | No difference | No difference | 3.399 |
| Bombyx mori myosin heavy chain (Mhc) | GI: 512902557 | 2.200 | 3.708 | 3.186 |
| C-type lectin 10 precursor | GI: 148298818 | No difference | No difference | 3.161 |
| Titin-like protein | GI: 18700461 | 3.174 | 2.215 | 3.143 |
| silkworm storage protein | GI: 379327811 | No difference | No difference | 3.080 |
| clip domain serine protease 11 precursor | GI: 112983100 | 2.507 | No difference | 3.031 |
| catalase | GI: 112982683 | No difference | No difference | 2.998 |
| odorant binding protein | GI: 226000891 | No difference | 1.7707 | 2.819 |
| heat shock protein 1 | GI: 148298768 | 2.8065 | 1.707 | 2.796 |
| 30K protein 11 | GI: 379046496 | No difference | No difference | 2.681 |
| troponin T transcript variant B | GI: 225346689 | 2.124 | 1.586 | 2.630 |
| muscle LIM protein isoform 1 | GI: 160333386 | 3.393 | 1.892 | 2.564 |
| miniparamyosin | GI: 221327579 | 3.331 | 1.072 | 2.553 |
| glutamyl-tRNA amidotransferase subunit B | GI: 114051415 | 1.096 | No difference | 2.412 |
| chymotrypsin inhibitor CI-8A | GI: 14028769 | No difference | No difference | 2.333 |
| cationic peptide CP8 precursor | GI: 119514503 | No difference | 1.014 | 2.244 |
| β-esterase 2 | GI: 195963357 | No difference | No difference | 2.209 |
| sex-specific storage-protein 2 precursor | GI: 124430725 | -1.172 | 1.172 | 2.188 |
| AGT | GI: 512930742 | No difference | No difference | 2.136 |
| serine-pyruvate aminotransferase | GI: 114052256 | No difference | 1.456 | 2.129 |
| apolipophorin III precursor | GI: 112983018 | No difference | No difference | 2.085 |
| troponin I transcript variant C | GI: 225346695 | 1.920 | No difference | 1.798 |
| xanthine dehydrogenase | GI: 2282473 | No difference | No difference | 1.202 |
| Bombyx mori elongation of very long chain fatty acids protein 7-like | GI: 512899895 | -7.220 | -13.595 | -7.712 |
| Vacuolar protein sorting-associated protein 35 like | GI: 512906339 | No difference | No difference | -3.677 |
| cuticular protein RR-2 motif 72 | GI: 290560924 | -2.609 | No difference | -3.661 |
| vesicle amine transport protein | GI: 153792203 | -11.355 | -1.183 | -3.473 |
| lactate dehydrogenase | GI: 156255210 | -3.135 | No difference | -3.047 |
| enoyl-CoA hydratase precursor 1 | GI: 87248109 | No difference | No difference | -2.876 |
| small nuclear ribonucleoprotein G | GI: 114050793 | -3.7 | -1.456 | -2.839 |
| bolA-like 3 | GI: 114051307 | -3.621 | No difference | -2.360 |
| 90-kDa heat shock protein | GI: 112983556 | -1.500 | No difference | -2.176 |
| tumor suppressor candidate 3-like | GI: 512914377 | No difference | No difference | -2.037 |
| peptidylprolyl isomerase B precursor | GI: 114052472 | No difference | No difference | -1.961 |
| calreticulin | GI: 28804517 | -1.577 | No difference | -1.802 |
| heterogeneous nuclear ribonucleoprotein K-like isoform X1 | GI: 512936106 | -1.723 | No difference | -1.791 |
| cytochrome c oxidase polypeptide IV | GI: 118918433 | No difference | No difference | -1.743 |
